# Supplementary material for: The Community Pharmacy as a Study Center for the Epidemiological Analysis of the Population Vaccination against SARS-CoV-2: Evaluation of Vaccine Safety and Pharmaceutical Service
Source: Pharmacy (Basel). 2024 Jan 20;12(1):16. doi: 10.3390/pharmacy12010016 (PMC10892165; doi:10.3390/pharmacy12010016)
Supplement: Supplementary file 1 [file pharmacy-12-00016-s001.zip › pharmacy-2770431-supplementary.pdf]

Table S1.

UK (English) EQ-5D-Y Paper Self-Complete modified
(sample version, v2.1)
Under each heading, please tick the ONE box that best describes your health TODAY.

MOBILITY (walking about)
I have no problems walking about 1 □□
I have some problems walking about 2 □□
I have a lot of problems walking about 3 □□

LOOKING AFTER MYSELF
I have no problems washing or dressing myself □□
I have some problems washing or dressing myself □□
I have a lot of problems washing or dressing myself □□

DOING USUAL ACTIVITIES (for example, going to school, hobbies, sports, playing, doing things with family or friends)
I have no problems doing my usual activities □□
I have some problems doing my usual activities □□
I have a lot of problems doing my usual activities □□

HAVING PAIN OR DISCOMFORT
I have no pain or discomfort □□
I have some pain or discomfort □□
I have a lot of pain or discomfort □□

FEELING WORRIED, SAD, OR UNHAPPY
I am not worried, sad, or unhappy □□
I am a bit worried, sad, or unhappy □□
I am very worried, sad, or unhappy □□

ARE YOU SATISFIED WITH THE VACCINATION SERVICE OFFERED BY THE PHARMACY?
Very □□
Enough □□
Little □□

ARE YOU MORE IN FAVOR OF A FOURTH DOSE OF VACCINE OR ANTIVIRALS THERAPY?

. . . .
